# Comprehensive Review of Fluorescence Applications in Gynecology

**DOI:** 10.3390/jcm10194387

**Published:** 2021-09-25

**Authors:** Joanna Polom, Leszek Kalinowski, Michele Diana, Manish Chand, Carmela Caballero, Sambor Sawicki, Karol Polom

**Affiliations:** 1 Department of Medical Laboratory Diagnostics-Fahrenheit Biobank BBMRI.pl, Medical University of Gdańsk, Marii Skłodowskiej-Curie 3a, 80-210 Gdańsk, Poland; asiabuks@gmail.com (J.P.); leszek.kalinowski@gumed.edu.pl (L.K.); 2 Biobanking and Biomolecular Resources Research Infrastructure Poland (BBMRI.pl), 80-211 Gdansk, Poland; 3 BioTechMed Centre, Department of Mechanics of Materials and Structures, Gdansk University of Technology, 80-233 Gdansk, Poland; 4 IRCAD, Research Institute against Digestive Cancer, 67-091 Strasbourg, France; michele.diana@ircad.fr; 5 ICube Lab, Photonics for Health, University of Strasbourg, 67-091 Strasbourg, France; 6 Department of General, Digestive and Endocrine Surgery, University Hospital of Strasbourg, 67-091 Strasbourg, France; 7 Department of Surgery and Interventional Sciences, GENIE Centre, University College London, University College London Hospitals, NHS Trust, 250 Euston Road, London NW1 2BU, UK; m.chand@ucl.ac.uk; 8 Breast International Group. 20, Rue de Bretagne/Bretagnestraat, B-1200 Brussels, Belgium; ca-ballero.surgery@gmail.com; 9 Department of Gynecology, Gynecological Oncology and Gynecological Endocrinology Medical University of Gdańsk, Marii Skłodowskiej-Curie 3a, 80-210 Gdansk, Poland; sam-bor.sawicki@gumed.edu.pl; 10 Department of Surgical Oncology, Medical University of Gdansk, Marii Skłodowskiej-Curie 3a, 80-210 Gdansk, Poland

**Keywords:** indocyanine green, image-guided surgery, fluorophore, fluorescence

## Abstract

Since the introduction of indocyanine green (ICG) as a fluorophore in near-infrared imaging, fluorescence visualization has become an essential tool in many fields of surgery. In the field of gynecology, recent new applications have been proposed and found their place in clinical practice. Different applications in gynecology were investigated, subcategorized, and overviewed concerning surgical applications and available dyes. Specific applications in which fluorescence-guided surgery was implemented in gynecology are described in this manuscript—namely, sentinel node biopsy, mesometrium visualization, angiography of different organs, safety issues in pregnant women, ureters visualization, detection of peritoneal metastases, targeted fluorophores for cancer detection, fluorescent contamination hysterectomy, lymphography for lower limb lymphedema prevention, tumor margin detection, endometriosis, and metastases mapping. With evolving technology, further innovative research on the new applications of fluorescence visualization in cancer surgery may help to establish these techniques as standards of high-quality surgery in gynecology. However, more investigations are necessary in order to assess if these innovative tools can also be effective to improve patient outcomes and quality of life in different gynecologic malignancies.

## 1. Introduction

In 2005, when Kitai et al. introduced fluorescence-guided surgery (FGS) for breast cancer sentinel node biopsy using indocyanine green (ICG), a new era of image-guided surgery [1] was initiated. Some new systems can even merge these fluorescent images with white light images and help to better orientate intraoperatively [2,3]. Currently, there are only five FGS contrast agents approved for clinical use by the American Food and Drug Administration (FDA), as well as by European Medicines Agency (EMA), i.e., fluorescein, ICG, methylene blue, and 5-alanine. Novel fluorophores are still under investigation through ongoing clinical trials. We have to wait for stronger evidence before their wider clinical use can be achieved.

This review summarizes currently available applications in gynecology using near-infrared fluorescence imaging with special attention given to specific clinical applications.

## 2. Sentinel Node Biopsy

In gynecologic malignancies, the concept of SNB is not new. Historically, vulvar cancer was one of the first malignancies for which this concept was evaluated [4,5]. In endometrial cancer, the most common gynecologic malignancy, two randomized clinical trials investigated the efficacy of this technique. However, these trials did not support the hypothesis of improved survival after lymphadenectomy in the early stage of the disease [6,7].

### 2.1. Endometrial Cancer

Even though radiocolloid and blue dye are the most commonly used tracers for SNB in a majority of cancers based on a recent survey among American gynecologic oncologists, ICG was most commonly used for SNB in endometrial cancer (97.3% of the responders) and in cervical cancer (92.5%) [8,9].

The first application of ICG for the SNB procedure in endometrial cancer was published by Furukawa et al., in which fluorescent nodes were detected in 83% of patients, with all cases found bilaterally [10]. No false-negative nodes were found. The first minimally invasive SNB in endometrial cancer was published by Rossi et al., with the detection of sentinel nodes in 85% of cases (17 patients) [11]. In 60% of cases, positive nodes were found bilaterally, with no false-negative cases. In a study that compared ICG with isosulfan blue (IB), the nodes were found with the naked eye in 77% of cases with IB and in 97% of cases when using the fluorescent properties of ICG [12].

In a meta-analysis by Ruscito et al., ICG was compared to radiocolloid, blue dye, or the combination of radiocolloid and blue dye found similar results in the bilateral or unilateral detection rate, as well as a similar false-negative rate [13]. However, when comparing ICG to a blue dye, the detection was significantly improved by 26.5% using fluorescence guidance, as found in a randomized study [14]. Additionally, in a multicenter study, the bilateral detection of sentinel nodes was 10.6% higher when using ICG versus blue dye [15]. In a meta-analysis by How et al., ICG improved the overall detection rate by 94%, as compared to blue dye (86%) or radiocolloid (86%). This trend was also observed in the case of bilateral detection but was not observed in the case of para-aortic SNB [16]. A high BMI was found to be accountable for difficulties in SNB [17]. In a publication by Jewell et al., the median BMI in which SNB was found under fluorescence guidance was 30.1 kg/m^2^ in comparison to 41.2 kg/m^2^, in which nodes were not found [17]. BMI also had an impact on unilateral or bilateral SNBs for patients with a median BMI of 34 kg/m^2^ and 29.6 kg/m^2^, respectively. In another study, BMI ≥ 30 was found to be accountable for a successful bilateral node mapping, and ICG was superior to IB among patients with a higher BMI [17].

We are waiting for the results of two clinical studies that investigate the role of ICG in endometrial cancer SNB—SELECT study and SENTIRAD (NCT04291612; NCT02598219). Survival data from these studies will provide evidence for the use of ICG in the case of early stage endometrial cancer.

### 2.2. Cervical Cancer

The first experience in cervical cancer sentinel node biopsy using ICG was published by Crane et al., in which a mixture of ICG and patent blue was injected for lymph node staining [18]. SNB fluorescence was observed in 60% of cases (6 patients). Once pelvic lymphadenectomy was performed, additional nodes (11 ones) showed fluorescence with one fluorescent node harboring metastases. No false-negative results were found, and SNBs were found bilaterally in half of the cases. The detection rate in tumors below 2 cm was 80% and only 40% in larger tumors.

An application of this procedure in cervical cancer is of the highest importance since up to 20% of patients with early cervical cancer present with lymph node metastases. The accurate detection of nodal disease impacts 5-year survival, which decreases from 92 to 64% for those with lymph node metastases [19,20]. The application of sentinel node biopsy may decrease the long-term morbidity rate related to lymphadenectomy that occurs in up to 20% of those undergoing the more radical procedure [21].

In a publication by van der Vorst et al., different concentrations of ICG mixed with human serum albumin (HSA) were investigated. However, no significant difference was found when analyzing different doses [22]. In another publication originating from this group, i.e., randomization between ICG alone or ICG mixed with HSA, they found that the sensitivity of fluorescent SNB was 83.3% with a negative predictive value of 92.3% [2]. In a randomized study (FLIM trial), ICG was compared with blue dye with more nodes identified with ICG than with blue dye, without any difference in the pathological confirmation of the nodes between both dyes [23]. In the meta-analysis by Ruscito et al. in 2016, SNB in endometrial and cervical cancers showed similar results between ICG and blue dye together with radiocolloid. However, 2 years later in a meta-analysis by Ulain et al. including eight analyzed studies, ICG versus a combination of all other tracers revealed higher unilateral and bilateral detection rates [24]. However, in the latter study, no difference was found in the overall rate of SNB detection [24]. Additionally, when HSA was mixed with ICG, it did not improve the detection rate, in comparison to ICG alone. In this meta-analysis also, no benefit was found when combining ICG with blue dye or using blue dye alone over ICG [24]. However, no conclusions were drawn concerning the volume and concentration of ICG that should be injected, ranging from 0.5 to 5 mg/mL with volumes ranging from 0.2 to 4.0 mL. Another issue that needs to be resolved is the extent of injection that varies from a two-quadrant injection to a four-quadrant one, as well as peritumoral injection [25]. All of the above-mentioned issues need to be standardized to obtain improved results in the future.

### 2.3. Vulvar Cancer

The first description of an ICG use in vulvar cancer SNB was presented by Crane et al. in 10 patients and showed that this technique was feasible but only in patients with a BMI below 25 kg/m^2^ [26]. It was associated with limited penetration of fluorescence through extensive adipose tissue in the groin. This issue was also highlighted in a publication by Prader et al., in which the authors also found that in the group with BMI > 30 kg/m^2^, sentinel nodes were found in 93.3% of patients when radiocolloid was used and in 86.7% of patients in the ICG group [27]. In a publication by Verbeek et al., if ICG was mixed with radiocolloid in 12 patients with early stage vulvar cancer, the detection rate of sentinel nodes was 100% [28]. In a publication by Soergel et al. studying 27 patients, 8 sentinel nodes were found with ICG but not when radiocolloid was used [29]. A higher rate of sentinel nodes detected via ICG in comparison to radiocolloid was also published by Broach et al. Mapping was performed among 96 patients with radiocolloid and ICG injected in the groin. In 14.6% of cases, nodes were seen only with ICG [30]. In a multicenter randomized trial including 48 patients that compared mixed ICG with radiocolloid with a standard radiocolloid and a blue dye, sentinel node identification using fluorescence was possible in 92.5% of cases and in 65.3% [31] of cases when a blue dye was used. Additionally, a successful sentinel node detection rate in the standard method was found in 92.1% of cases and in 97.2% of cases in the fluorophore group. Another valuable aspect from this trial was that statistically significant more short-term postoperative complications were described in the standard group. A worthwhile application of ICG sentinel node biopsy was presented using a video endoscopic inguinal lymphadenectomy (VEIL) with sentinel node mapping using a robotic system [32].

### 2.4. Ovarian Cancer

In the systematic review from 2019, 10 articles were analyzed with a detection rate of 90.3% [33]. Concerning ICG and radiocolloid in seven ovarian cancer patients, the detection rate was 100% [34]. In a series of five patients using only ICG, sentinel nodes were detected in all cases [35]. Recently, published preliminary results of the prospective multicenter SELLY study, detection of sentinel nodes using ICG was 67.7%, with a much higher detection rate of patients who underwent immediate staging [36]. Four patients presented with lymph node metastases, and all four were identified with ICG. These data were lower than the ones previously published in the review article in which the detection rate was found to be 88% [37]. Further research is required to show the real potential of fluorescent tracers in SNB procedures in ovarian cancer.

### 2.5. Vaginal Cancer

In this malignancy, only a case report presenting a successful sentinel node mapping in vaginal cancer is available [38]. ICG was injected into the bilateral side of the tumor. Sentinel nodes were found bilaterally in the obturator fossa.

## 3. Multi-Channel Fluorescence

The issue of the injection site in uterine cancer or of the cervical injection in endometrial cancer is still debatable. Sentinel nodes might be visible using two fluorophores during the same operation. In a study by Laios et al., the fluorescence properties of methylene blue were used. Methylene blue and ICG were visible in two patients sharing common lymphatic structures no matter the injection site [39]. In one case, the lymph nodes were stained with both fluorophores. However, in the second case, the true positive para-aortic sentinel node was only stained with ICG after uterine fundus injection but not with MB after cervical injection. Similar multispectral fluorescence imaging during prostatectomy was proposed using fluorescein and ICG mixed with radiocolloid [40]. Undoubtedly, multispectral image-guided surgery will soon be a perfect tool to differentiate anatomical structures from different fluorophores.

## 4. Mesometrium

Enhancements in surgical techniques that will help to improve oncologic outcomes are among the key objectives of any surgical research. Total mesorectal excision (TME) proposed by RJ Heald revolutionized the surgical technique for several cancers, as well as the concept of embryological planes. This led to an improved local control after rectal cancer surgery [41]. This idea was also adopted in other organ-specific surgical techniques [42,43]. Embryologically based compartmental surgery was also proposed by Höckel et al., in the treatment of gynecologic cancer [44]. In the publication by Kimmig et al., the visualization of compartments was supported by the injection of ICG into the uterine corpus [45]. Lymph nodes, as well as complex lymphatic vessels, can be observed. Therefore, the whole organ compartment can be seen with fluorescence. This intraoperative lymphography revealed two pathways of lymphatic flow, the first one along the uterine vessels to the iliac lymph nodes in the pelvis, and the second one along the ovarian vessels to the para-aortic lymph nodes [46,47,48].

## 5. Fluorescent Angiography

### 5.1. Uterine Tube Perfusion

The concept of uterine transplantation is emerging, although it is still in its infancy period. During donor hysterectomy, normally, both uterine tubes are transected [49]. In the case of pregnancy, the recipient of the uterus has to undergo an in vitro fertilization (IVF). It is hypothesized that transplantation made together with the uterine tubes may facilitate a spontaneous conception [50]. In the study by Farag et al., they investigated ex vivo and in vivo relative fluorescence, as well as the fluorescence intensity ratio [51], using ICG angiography. Vascular perfusion for uterine tubes originates from utero-ovarian vasculature alone. This is especially important since a less extensive dissection for bilateral arterial and veins is currently proposed during transplantation [52]. Additionally, the authors found differences in the location of utero-ovarian vessels. The length of utero-ovarian vessels may also help with an easier re-anastomosis and a safer surgery. This study may pave the way for complex uterus and attached uterine tubes transplantation, based on a tailored separation of utero-ovarian vasculature with fluorescence guidance, and facilitate a spontaneous conception.

### 5.2. Ureteral Branch of Uterine Artery Detection

The surgical treatment of cervical cancer requires an extensive conventional radical hysterectomy, which is sometimes associated with a poor blood supply of the ureters postoperatively. This may lead to complex postoperative complications such as ischemic necrosis of the ureter, urinary fistula, or stenosis of the ureter [53,54]. Possible prevention of such complications might be proposed by preserving the ureteral branch of the uterine artery [54]. A report of two cases with ureteral branch-sparing hysterectomy was presented using fluorescence angiography for the identification of a ureteral branch [55]. Additionally, with this fluorescence angiography technique, the authors also evaluated the perfusion of the uterine artery and ureter. No postoperative complications related to the ureter vasculature were reported.

### 5.3. Vaginal Cuff Angiography

Vaginal cuff dehiscence represents a severe complication after hysterectomy. The implementation of robotic surgery increased the rate of this complication, with a rate of 0.6% for abdominal hysterectomy and up to 3.16% after robotic hysterectomy [56]. Vaginal cuff angiography using ICG was performed after robotic hysterectomy in 20 patients [57]. No difference in terms of vaginal cuff dehiscence prevention was observed for monopolar or ultrasonic devices. Only longer instrument activation times in the open cuff showed a decreased cuff perfusion. A similar study was performed for laparoscopic hysterectomy [58]. The added value of ICG guidance to vaginal cuff angiography and flap reconstructing is improving the technique, as it is now used in daily practice. Then, another point is that it helps to decrease the morbidity rates.

Future studies of this method to decrease postoperative complications will show its clinical relevance, especially in robotic surgery.

### 5.4. Flap Reconstruction

Postoperative complications after bilateral groin lymphadenectomy is not a rare situation. However, a wound breakdown is a difficult complication to treat. In the literature, a case was reported with such a complication, and a pedunculated left anterolateral thigh flap was presented [59]. A fluorescence angiography with ICG for flap viability was performed without any further complications associated with flap healing. Two months after the operation, the patient received radiotherapy.

### 5.5. Uterus Transplantation

From a surgical standpoint, the key to successful uterus transplantation is the quality of vascular anastomoses. An occlusion of anastomoses is complex and might be associated with inadequate anticoagulation, poor graft fixation, low immunosuppression, inadequate surgical technique, patient’s age, and mutations associated with venous thromboembolism [60]. Kengelbach et al. used different techniques in a sheep model for intraoperative and postoperative blood flow measurements using ICG, as well as Doppler flowmetry [60]. A similar study was performed in four cynomolgus macaques with an evaluation of uterine artery and vein anastomoses [61]. Both methods proved to be useful for uterus vascularization, as well as for the patency of vessels. In a publication of Obara et al. in four cynomolgus macaques, allogenic uterus transplantation was performed [62]. ICG fluorescence angiography showed adequate blood flow to all transplants. In one case, fluorescence angiography showed blood flow from the left to the right side of the uterus. In another study, six cynomolgus macaques were evaluated after unresponsive immunosuppression [63]. They analyzed all different clinicopathological factors associated with transplant rejection. An ICG fluorescence imaging of all transplanted grafts showed a swollen uterus without fluorescence of this organ. Another study evaluated the number of vessels necessary for uterine blood flow in animal models [64]. They found that even one uterine artery is sufficient to visualize the fluorescence of the uterus. Additionally, ovarian vessels did not show any significance related to uterine fluorescence. However, when uterine arteries were still clamped via the ovarian arteries, it prolonged the time to reach perfusion at its maximum. In pregnant macaques with only the right uterine artery and vein, fluorescence using ICG was performed [65]. In the third trimester, fluorescence imaging was performed, and under near-infrared light, a uterine body was visualized from the right to the center with additional collateral circulation, starting from the right artery next to the left uterine artery, showing the left part of the uterine body at the end.

### 5.6. Trachelectomy and Uterine Artery Angiography

Trachelectomy is performed in early stage cervical cancer among patients desiring future fertility. There is some extensive literature evidence regarding its safety, as well as good outcomes associated with 16 to 23% of the pregnancy rate. However, one of the questions associated with surgical steps is the preservation of the uterine artery during this operation. In the group of 20 patients, half of them underwent uterine artery sparing trachelectomy, and the second half, uterine non-sparing surgery [66]. A uterine fluorescence with ICG was measured for both groups. No difference in fundal fluorescence perfusion was found between the two analyzed groups, which proved that uterine viability is not associated with uterine artery preservation during trachelectomy.

### 5.7. Intestinal Angiography for Gynecology

The role of ICG fluorescence angiography for colorectal surgery is well documented [67]. In an article describing 100 consecutive bowel anastomoses for gynecologic malignancies, ICG was used for colon angiography [68]. For low anterior resections (LARs), an endoscopic camera system was used transanally, and for other types of anastomoses, a handheld system was used. ICG angiography has led to two anastomotic revisions and one diverting ileostomy [68]. There was one postoperative leakage. In a case report of a rectosigmoid endometriotic nodule and an intravenous ICG angiography of the ischemic area around the rectal lesion, a transection line was selected based on this examination [69]. In a publication by Bourdel et al. studying a group of 21 patients who underwent deep infiltrating endometriosis (DIE) resection, ICG angiography was used for vascularization checkup after rectal shaving [70]. Adequate fluorescence of this area was possible in 81% of cases. In one patient, two stitches were made to reapproximate the rectal muscularis layer, which improved the fluorescence of this area. No postoperative fistula was diagnosed. A valuable application of ICG angiography in gynecology using laparoscopic intestinal angiography was presented during intestinal vaginoplasty for intestinal segment perfusion in six transgender patients [71]. In one patient, fluorescence angiography was inadequate, and vaginoplasty was aborted.

## 6. Other Major Applications of ICG

### 6.1. ICG Safety in Pregnant Women

The safety of ICG use in pregnant women is still not entirely investigated. It was shown that ICG was not found in fetal cord blood or in umbilical vein blood just after birth [72]. It was demonstrated that ICG was found in the fetus in mouse models. In addition, drugs can increase their distribution to the fetus [73]. The study that analyzed an impact of ICG transplacental transfer in ex vivo perfusion models from cesarean deliveries showed a minor fetal reservoir of ICG [74]. Additionally, they found that it was probably mediated by means of an organic anion transporting polypeptide (OATP). It seems that the placenta is a protective barrier for ICG spread to the fetus even though it is not a full barrier.

### 6.2. Ureteral Visualization

Ureter injury during surgery is a rare event. However, it is considered to be one of the most severe postoperative complications. After the extensive resection of the ureteral endometrial masses, the local perfusion of the ureter was examined in 31 ureters [75]. Local ischemia was suspected in five cases (16.1%). However, in three cases, irregular fluorescence or absent fluorescence was observed, and stent placement was administered. No postoperative complications were noted.

Another valuable idea is to administer intraurethral ICG via a cystoscopic catheter. In a group of 30 operations, the preoperative procedure of cystoscopy prolonged time over approximately 7 minutes and in 10 patients (33%); in case of difficulties to intraoperatively find the ureter in normal light, the fluorescence-guided identification made it possible to find its position faster [76]. In a group of 16 gynecologic oncology operations, all bilateral ureters were visualized after the cystoscopic insertion of 8 mL of ICG with 6 French ureteral catheters [77]. Fluorescence visualization of the ureters and the bladder was used during neovagina creation in congenital vaginal agenesis [78]. In a group of four patients, ICG was injected into both ureters through six French catheters. This technique seems to be effective. However, the fact that more time is needed during an operation, and that an invasive catheter insertion is performed, leading to the risk of iatrogenic ureteral injury, are disadvantages of this method.

Methylene blue is another dye presenting fluorescence properties, which can be used to visualize ureters. The first description of methylene blue visualization in the ureters during a colorectal operation after intravenous injection was published by Verbeek et al. [79]. In addition, other investigators described the use of this dye for the fluorescent search for ureters [80,81,82].

New drugs are investigated to visualize ureters in fluorescence. One of these has been recently used in a phase 1 study during a hysterectomy (IS-001 [83]). The ureters were visualized in all investigated patients. In 41 patients, nerindocianine sodium was investigated as another drug, and fluorescence of the ureters was found in 88.9% of cases [84]. In a recent systematic review of clinically available and experimental dyes for ureteral visualization in near-infrared light in laparoscopic surgery, it was found that methylene blue was better than ICG, and additionally, ZW800-1 showed greater fluorescent properties, making it a promising fluorophore in the future [85].

### 6.3. Peritoneal Metastases

Due to difficulties in finding tiny nodules in the whole abdomen during cytoreductive surgery, it is sometimes impossible to resect all metastatic nodules. In a group of 10 patients, 20 mg of ICG, administered intravenously after opening the abdominal cavity, was used for the fluorescent detection of suspected gynecologic cancer [86]. Only six patients had a malignant disease, only two had peritoneal metastases, and eight metastatic lesions were found under near-infrared light. The authors highlighted the fact that 13 non-malignant lesions were also found, reaching a false positive rate of 62%. Additionally, as they analyzed signal-to-background ratios between malignant and non-malignant nodules, no difference was found (2.0 vs. 2.0). In a recent meta-analysis of ICG use in the detection of peritoneal metastases in different cancers, sensitivity varied from 72.4 to 100% and specificity from 54.2 to 100% [87].

### 6.4. Targeted Fluorophores for Cancer Detection

Another step forward in the detection of a malignant tumor and its metastases are targeted fluorescent probes that may specifically bind to cancer antigens and be detected by adjacent fluorophores. Surgical tumor margins, metastatic lymph nodes, peritoneal and other solid organ metastases might be seen intraoperatively in fluorescent light [88]. In a clinical model, five most prominent targets in ovarian cancer were proposed: folate receptor α, vascular endothelial growth factor, epidermal growth factor receptor, chemokine receptor 4, and matrix metalloproteinase. The first-in-human trial showed a possible potential in tumor-specific fluorescence of ovarian cancer using FR α targeted agent folate FITC [89]. In peritoneal carcinomatosis, a tumor lesion detected when using tumor-specific fluorescence, a median of 34 vs. 7 lesions was found when using the naked eye only [89]. An OTL-38 was used in a preliminary study in four patients with serous or clear cell endometrial cancer [90]. Lymph node metastases and omental lesions were detected with targeted fluorescence. An interesting OTL-38 -a fluorescent probe that targets folate receptor α allowed the identification of 29% more metastatic lesions during surgery for ovarian cancer in 12 patients when compared to standard clinical inspection and or palpation [91]. However, 11 out of 13 lymph nodes stained with this fluorophore were tumor-free after pathological examination [91].

Other targets were investigated by the same group, and an expression of EpCAM showed a similar expression as folate receptor α in peritoneal metastases and was not found in lymph nodes [92]. Here, we have to point out that EpCAM, as well as folate receptor α, represent a high expression in epithelial cells of tumor negative uterine tubes and uterine endometrial cells, which may interfere in early stage cancer detection in the future [92]. Undoubtedly, this field is rapidly evolving and, in the future, we will probably have a few specific targeted dyes for different cancers in gynecology.

### 6.5. Fluorescent Contamination Hysterectomy

Minimally invasive surgery is currently receiving more attention in gynecology. However, in two randomized studies on cervical cancer, the minimally invasive approach was associated with worse disease-free survival, as well as with overall survival [93,94]. The reason behind this is still unknown and an intracorporeal colpotomy during radical hysterectomy might be accountable for the intraperitoneal dissemination of tumor cells. An interesting study was published that used ICG to show hypothetical contamination of cervical secretion caused by manipulation during intracorporeal colpotomy [94]. After ICG application to the cervical surface, minimally invasive surgery was performed [95]. In 12 patients, peritoneal contamination was found in nine patients (75%). Additionally, in seven patients (58%), contamination of laparoscopic instruments was found. Undoubtedly, other factors may also play a role in achieving worse results after a minimally invasive hysterectomy. However, this application of ICG may serve in the future as a quality assessment tool of peritoneal contamination with cervical secretion.

### 6.6. Lymphography to Prevent Lower Limb Lymphedema

Lower limb lymphedema is one of the most difficult postoperative complications to treat after an extended lymphadenectomy in small pelvis cancer surgeries [96]. In lower limb lymphedema, Yamamoto et al. described three patterns of lymphedema after ICG injection: splash pattern, stardust, and diffuse pattern [97]. In a group of 68 patients, 37 of them developed lower limb lymphedema after cervical cancer treatment [98]. The splash pattern at the groin level was found to be present in patients with clinically reduced lymphedema, whereas significant lymphedema was found in a diffuse pattern on the whole length of the lower limb. A possible lymphaticovenular anastomosis for lower limb and genital lymphedema was found in case of severe lymphorrhea [98]. Another possibility to treat lower limb lymphedema is to harvest right supraclavicular lymph nodes with lower limb node flap transfer in 10 patients, with 6 cases after endometrial cancer lymphadenectomy [99]. ICG lymphography was performed to visualize lower limb lymphatic mapping, as well as the supraclavicular area. An effective decrease in lower limb lymphedema was reported in all patients.

### 6.7. Margin Detection

The real-time detection of tumor margins may help to achieve a better complete resection. Two case reports presented an interesting use of ICG in vulvar and vaginal cancer for surgical resection guidance [100]. In the first case after primary incomplete resection of vulvar cancer, an intravenous ICG injection determined the free margin after resection. In the second case, peritumoral ICG injection 1 cm around the upper vagina cancer showed a clear resection margin on the final pathological finding [100]. Both methods need to be evaluated on a larger scale. However, they seem to be valuable techniques for an image-guided surgery approach in gynecology [100].

### 6.8. Endometriosis

The ICG use for endometriosis detection is made possible via the intraoperative visualization of tissue vascularization. In a group of 27 patients in a pilot study of the Gre-Endo trial, 100 lesions were seen in white light, and an additional 16 lesions were found under fluorescence guidance [101]. In a group of 63 patients, 173 lesions were excised, and 90.4% of them were confirmed to be histologically proven endometriosis [102]. A total of 166 (96%) lesions were found with the naked eye and 32 (18.5%) lesions with ICG [102]. Here, 22 lesions proved to be endometriosis. Only seven additional lesions were identified with ICG, and only one proved to be endometriosis. In a systematic review based on 17 studies, 8 lesions showed the usefulness of ICG for endometriosis detection. One randomized trial, and one prospective study failed to show any advantage of ICG in endometriosis detection. Eight other studies showed the usefulness of ICG in the evaluation of intestinal angiography anastomoses, as well as ureterolysis in deep infiltrating endometriosis [103]. The use of ICG was shown to be effective in the case of segmental resection of deep infiltrating endometriosis of the rectosigmoid part of the colon [103]. In a publication by Raimondo et al., a correlation between vascular patterns of rectosigmoid endometriosis was found in 30 patients with a hypovascular pattern (60%) in larger bowel endometriosis [69]. The potential use of artificial intelligence (AI) in intraoperative tissue classification with ICG perfusion of colorectal cancer was recently published, and this idea might be also translated into endometriosis evaluation [104].

In a publication by Lier et al., 3D imaging showed significantly better sensitivity and a non-inferior specificity in comparison to 2D standard imaging [105]. The additional use of narrow-band imaging (NBI) or ICG-based FGS showed no improvement in endometriosis detection [105].

In endometriosis FGS, it has to be stressed that previous retroperitoneal surgery, fibrosis, reduction of neo-angiogenesis with the use of estrogen–progestin, or gonadotropin-releasing hormone agonist might alter microcirculation and ultimately endometriosis detection [102,106,107].

Potential ureteral microcirculation after ureterolysis was already described in Section 6.2 “Ureteral Visualization”.

### 6.9. Metastases Mapping

In three patients with metastatic-suspected lesions caused by cervical cancers or carcinosarcoma found on PET/CT-scan, a tomography-guided percutaneous injection of ICG was performed, and the lesions were then identified during laparoscopic fluorescent visualization [108]. Such a technique might be used for difficult-to-reach or small lesions suspected of malignancy.

## 7. Conclusions

Fluorescence-guided surgery also found a well-established place in gynecology. This image-guided surgery offers real-time control of the operative view with a clear benefit for the surgeon. We outlined an extensive review of currently available applications in gynecology, showing that this field also uses this modern technology for a variety of adopted strategies. Undoubtedly, for wider use of this technology, we will need large-scale, well-designed trials, which will prove the necessary evidence of possible applications of this technology.
